# Preparation and Immunogenicity Prediction of *Brucella melitensis* mRNA Vaccine Candidate Based on *omp16* and *omp19* Genes

**DOI:** 10.3390/vaccines14030240

**Published:** 2026-03-05

**Authors:** Jingjie Zhang, Haiyan Borijihan, Yixuan Chen, Huricha Baigude, Lili Bao, Fu Quan, Dezhi Yang

**Affiliations:** 1 Department of Infectious Diseases, Affiliated Hospital of Inner Mongolia Medical University, Hohhot 010110, China; jingjie0913@126.com; 2 National and Local Joint Engineering Research Center of Modern Mongolian Medicine Research and Testing, International Mongolian Hospital of Inner Mongolia, Hohhot 010065, China; haiyan2020222@163.com (H.B.); cyx2047259245@163.com (Y.C.); hbaigude@imu.edu.cn (H.B.); 3 School of Chemistry & Chemical Engineering, Inner Mongolia University, Hohhot 010020, China; 4 Department of Basic Medicine, Inner Mongolia Medical University, Hohhot 010110, China; 5 Department of Clinical Laboratory, Affiliated Hospital of Inner Mongolia Medical University, Hohhot 010110, China

**Keywords:** *Brucella melitensis*, *omp16*, *omp19*, epitope, mRNA vaccine

## Abstract

Background: *Brucella* outer membrane proteins (Omps) are an important part of its cell wall and major virulence-related factors. Omp16 and Omp19 proteins are the advantageous antigens of *Brucella* and have been widely used in research on vaccines against brucellosis. As an emerging vaccine, the mRNA vaccine has unique advantages in the fight against intracellular parasitic bacteria. Methods: In this study, mRNA encoding the *omp16* and *omp19* genes of *Brucella. melitensis* (*B. melitensis*) was synthesized using in vitro transcription. The target mRNA was transfected into HEK 293T cells to evaluate protein expression levels and assess its immunogenicity. Finally, bioinformatic approaches were employed to analyze potential antigenic epitopes. Results: In this study, the successfully constructed recombinant plasmids pIVTRup-*omp16* and pIVTRup-*omp19* were utilized to synthesize *omp16*-mRNA and *omp19*-mRNA, each approximately 600 nt in length. Western blot analysis detected the expression of proteins with molecular weights of 16 kDa and 19 kDa in HEK 293T cells at 24 h post-transfection with mRNA. Purified rOmp16 and rOmp19 had good immunogenicity, which could specifically bind to serum antibodies of brucellosis patients. rOmp16 had stronger immunogenicity than rOmp19. Epitope prediction showed that Omp16 contained seven epitopes and Omp19 contained six epitopes. In addition, Omp16 and Omp19 could form stable complexes with target receptors. Simulated immunization with Omp16 and Omp19 proteins significantly activated both CD4^+^ and CD8^+^ T cells. Conclusions: The immunogenic proteins were successfully expressed in cells based on the mRNA fragments synthesized from *omp16* and *omp19* genes of *B. melitensis*, which was a preliminary exploration for the preparation of *B. melitensis* mRNA vaccine.

## 1. Introduction

*Brucella* is an intracellular parasitic, globular, Gram-negative bacterium that infects ruminants and humans, causing brucellosis [1]. Recent studies show that there are 16,000 to 2.1 million new infections each year, which has a huge impact on population health and animal husbandry development [2]. *Brucella melitensis* (*B. melitensis*) is the most common and most pathogenic species of *Brucella* [3]. Goats and sheep are considered the natural hosts for *B. melitensis*, and infection often leads to impaired fertility [4]. Humans typically become infected through contact with infected animals or consumption of contaminated animal products, resulting in a chronic debilitating febrile illness [5,6]. The incubation period for human brucellosis generally ranges from 5 to 60 days, with common clinical manifestations including fever, profuse sweating, fatigue, arthralgia, and generalized myalgia [7]. Vaccination remains the primary strategy for preventing and controlling its transmission. Attenuated live vaccines, such as S19, RB51, SR82, and Rev1, are the most common types developed for livestock [8]. This highlights an urgent need for novel, safe, and effective vaccine strategies.

With the advancement of precise molecular technologies and an improved understanding of brucellosis pathogenesis, novel genetically engineered vaccines have been developed to replace conventional ones for the prevention and control of brucellosis [9,10]. In recent years, nucleotide-modified messenger RNA (mRNA) vaccines have emerged as a leading platform with great potential against a wide range of infectious pathogens [11]. Unlike DNA vaccines, mRNA is translated directly in the host cytoplasm without the need for nuclear entry or genomic integration, and is eventually degraded, thereby reducing the risk of genetic dysregulation [12].

The selection of an appropriate antigen is paramount for vaccine efficacy. The outer membrane proteins (Omps) family maintains bacterial integrity while eliciting a strong immune response and is thought to be involved in *Brucella* survival and immune escape in macrophages [5,13]. Among these, Omp16 and Omp19 lipoproteins are highly conserved across major pathogenic *Brucella* species and are considered advantageous antigens for brucellosis vaccine development [14,15]. Omp16 has been shown to induce strong Th1-biased immune responses, while Omp19 plays a vital role in mucosal immunity by interacting with Toll-like receptor 2 (TLR2) [16,17,18,19]. Therefore, Omp16 and Omp19 represent ideal targets for preparing brucellosis vaccines.

This study aims to utilize the key antigens Omp16 and Omp19 from *B. melitensis* to construct an in vitro transcription vector for generating antigen-encoding mRNA. We will investigate their protein expression in eukaryotic cells and assess the immunogenicity of the expressed proteins. Furthermore, bioinformatic approaches will be employed to predict and analyze B-cell and T-cell epitopes of *B. melitensis* Omp16 and Omp19 proteins. Molecular docking with corresponding receptors and immune challenge simulations will be conducted to preliminarily evaluate the feasibility of an mRNA-based vaccine against *Brucella*, thereby providing a theoretical foundation for the development of novel brucellosis vaccines.

## 2. Materials and Methods

### 2.1. Plasmids, Strains and Cells

pIVTRup plasmid was purchased from Addgene Company (Cambridge, MA, USA). pCDNA3-EGFP plasmid was preserved by Innovation Mongolian Medicine Engineering Research Center of Inner Mongolia International Mongolian Medical Hospital (Hohhot, China). Competent cells *E. coli* TransT1 were purchased from Beijing Transgen Biotech (Beijing, China). Human embryonic kidney 293T cells (HEK 293T) (ATCC: CRL-3216) were cultured in DMEM medium containing 10% fetal bovine serum (Gibco, Waltham, MA, USA), 100× streptomycin mixture (Solarbio, Beijing, China) and cultured in a constant temperature incubator containing 5% CO_2_ at 37 °C.

### 2.2. Liposomes, Brucella Genome and Serum

Liposome Dogo 4 was provided by Professor Huricha Baigude, School of Chemical Engineering, Inner Mongolia University [20]. *B. melitensis 6144* genome (Genbank: CP098767.1) and brucellosis patients (10 cases) and healthy human serum (10 cases) were from Clinical Laboratory Center, Affiliated Hospital of Inner Mongolia Medical University. Positive serum inclusion criteria: ① clinical diagnosis of brucellosis; ② *Brucella* serum antibody test positive, antibody titer >1:50; ③ fever, fatigue and lumbago and other obvious clinical symptoms. Negative serum was collected from the healthy check-up population, with the criterion: negative for *Brucella* antibody testing.

### 2.3. Construction of Plasmid pIVTRup-omp16/omp19/EGFP

Search Genbank for *B. melitensis omp16* and *omp19* gene sequences (*omp16* GeneID: 29593101; *omp19* GeneID: 29594621), and the *EGFP* gene sequence on pCDNA3-*EGFP* plasmid. Primer 5.0 software was used to design primers containing Flag tag sequences (Table 1). The *omp16*, *omp19* and *EGFP* gene fragments purified by agarose gel were cloned into the in vitro transcription vector pIVTRup to obtain pIVTRup-*omp16,* pIVTRup-*omp19* and pIVTRup-*EGFP.*

### 2.4. Synthesis of Target mRNA

#### 2.4.1. DNA Template

pIVTRup-*omp16* and pIVTRup-*omp19* were digested with *Eco*R I (NEB, Ipswich, MA, USA) and linearized respectively. pIVTRup-*EGFP* was amplified by PCR to obtain DNA template of suitable fragment. *EGFP*-F and M13R were used as upstream and downstream primers to prepare amplification reaction system with volume of 50 μL. The reaction conditions: (95 °C, 30 s; 55 °C, 30 s; 72 °C, 1 min, 20 s; 35 cycles in total); 72 °C, 5 min; 4 °C. DNA template for in vitro transcription was purified as described above using Agarose Gel DNA Extraction Kit (TaKaRa Bio, Kusatsu, Japan).

#### 2.4.2. In Vitro Transcription

HiScribe^®^ T7 ARCA mRNA in vitro transcription kit (NEB, Ipswich, MA, USA) was used to prepare transcription system in EP tube according to Table 2. 37 °C water baths for 30 min, 1 µL DNase I was added, and after mixing, 37 °C water bath for 15 min to remove DNA template. Target mRNA was purified with LiCl and ethanol according to kit instructions and stored at 80 °C.

#### 2.4.3. Target mRNA Identification

A 5% denaturing PAGE gel was prepared and pre-electrophoresis was performed in 1 × TBE electrophoresis buffer at 200 V for 30 min. A 2 µL RNA sample and a 2 µL RNA Ladder were mixed with 2 µL loading buffer respectively and placed into PCR instrument at 95 °C for 5 min, then placed on ice rapidly for 5 min to prevent RNA renaturation. RNA sample was added into gel well and electrophoresis was performed at 80 V for 25 min until bromophenol blue reached the bottom of gel. The gel block was soaked in nucleic acid dye for 1 h and the results were observed under an ultraviolet lamp.

### 2.5. HEK 293T Cell Transfection

HEK 293T cells were inoculated into 10 mm cell culture dishes and cultured at 37 °C for 16 h in 5% CO_2_ until the cell fusion reached 80%. The cells were replaced with DMEM basal medium. 20 µL Dogo 4 liposome and 500 µL Opti-MEM (Gibco, Waltham, MA, USA) were added to each enzyme-free EP tube, mixed and allowed to stand at room temperature for 5 min to prepare solution A. In new enzyme-free EP tubes, 20 µL ddH_2_O, 20 μg *omp 16*-mRNA, *omp19*-mRNA and *EGFP*-mRNA were mixed with 500 µL Opti-MEM serum reduced medium to prepare solution B. Solution B was added to solution A and incubated at room temperature for 5 min. Solution AB was added slowly to the cells and cultured at 37 °C for 5 h in 5% CO_2_, then replaced with DMEM complete medium containing 10% fetal bovine serum and 1% streptomycin for 19 h. Green fluorescence of *EGFP*-mRNA group was observed under an fluorescence microscope 24 h after transfection.

### 2.6. Extraction and Purification of Protein

Twenty-four hours after transfection, *omp16*-mRNA, *omp19*-mRNA and *EGFP*-mRNA groups were collected into EP tubes with 1 × PBS, and the supernatant was discarded. 700 μL RIPA lysate (Beyotime, Shanghai, China) containing 1 mM PMSF (Beyotime, Shanghai, China) was added to each tube to lyse the cells. Purify the protein of interest using Pierce™ Anti-DYKDDKAffinity Resin (Thermo Fisher Scientific, Waltham, MA, USA), collect the first wash solution (containing the internal reference protein) into EP tubes, store the final eluate (containing the protein of interest), and freeze at 80 °C.

### 2.7. Western Blot Analysis of Protein Expression

A total amount of 30 µL of purified protein samples was mixed with 6 µL of 6× Protein Buffer, heated at 95 °C for 5 min, and placed on ice. Western blotting was performed according to the instructions of SDS-PAGE gel preparation kit (CWBio, Taizhou, China). Flag-Tag Mouse Monoclonal Antibody (Absin, Shanghai, China) was used to detect Omp16 and Omp19 proteins expressed by transfected cells. GAPDH Mouse Monoclonal Antibody (Absin, Shanghai, China) was used to detect internal reference proteins. FITC-conjugated Goat Anti-Mouse IgG (H + L) was used as secondary antibody. The results were observed with a two-color infrared laser imager (Odyssey CLX, 700 nm and 800 nm).

### 2.8. ELISA to Detect Protein Immunogenicity

To detect protein immunogenicity, 0.5 μg purified rOmp16 and rOmp19 protein coated 96 well plate respectively. PBST washed 3 times, each well added 200 µL PBST preparation of 1% skim milk powder blocked at room temperature for 1 h. PBST washed 3 times, each well added 100 µL diluted brucellosis patient serum, control group added healthy human serum, incubated at 37 °C for 1.5 h. PBST wash buffer 4 times, add 100 µL HRP-labeled rabbit anti-human IgG (1:5000) to each well, incubate at 37 °C for 1 h. PBST wash buffer 5 times. Soluble TMB kit (CWBio, Taizhou, China), adding 100 µL of stop solution per well (1 mol/L HCL), and after 15 min, the OD value at 450 nm was read using a microplate reader. The results of rOmp16 and rOmp19 reactions with serum were expressed as mean ± standard deviation of OD_450_. The experiment was repeated three times. The experimental data were plotted using GraphPad Prism 9.5 software. Statistical differences in serum immune response between patients and healthy subjects were analyzed by independent sample *t*-test, and statistical differences in immunogenicity between rOmp16 and rOmp19 were analyzed by paired sample *t*-test. *p* < 0.05 was considered a statistical difference.

### 2.9. Prediction of Antigenic Epitopes of Omp16 and Omp19 Proteins

#### 2.9.1. Prediction of B Cell Epitopes for Omp16 and Omp19 Proteins

Comprehensive prediction of B-cell epitopes for Omp16 and Omp19 proteins was performed using the online prediction software IEDB (Bepipred Linear Epitope Prediction 2.0), ABCpred (https://webs.iiitd.edu.in/raghava/abcpred (accessed on 23 May 2025), and the SVMTriP database (http://sysbio.unl.edu/SVMTriP (accessed on 24 May 2025) [21,22].The response threshold criteria for the three databases were set at 0.5, 0.8, and 1.0, respectively.

#### 2.9.2. Prediction of T Cell Epitopes for Omp16 and Omp19 Proteins

Integrated prediction of T-cell epitopes for Omp16 and Omp19 proteins was performed using the online prediction software IEDB and the SYFPEITHI database (https://syfpeithi.de/) (accessed on 25 May 2025) [23,24,25]. Major histocompatibility complex (MHC) molecules are categorized into class I and class II, which activate cytotoxic T lymphocytes (CTL) and helper T lymphocytes (HTL) in the human body, respectively. Based on a list of common and well-documented human leukocyte antigen (HLA) alleles in the Chinese population, four alleles encoding MHC class I molecules (HLA-A*02:01, HLA-A*11:01, HLA-A*24:02, HLA-C*07:02) and four MHC class II alleles (HLA-DRB1*03:01, HLA-DRB1*07:01, HLA-DRB1*09:01, HLA-DRB1*15:01) were selected [26].Peptides with an IC50 value < 500 nmol/L predicted by NetMHCpan 3.0 and an immunogenicity score > 0.2 for MHC-I were identified as CTL epitopes. HTL epitopes were screened using NetMHCIIpan 4.0 from the IEDB database by selecting peptides ranked within the top 0.2%. The SYFPEITHI database was applied to predict both CTL and HTL epitopes, and peptides with a score > 20 were selected.

### 2.10. Prediction of Epitope Allergenicity and Toxicity

The toxicity of each epitope was assessed using the ToxinPred server (https://webs.iiitd.edu.in/raghava/toxinpred/ (accessed on 29 May 2025), while allergenicity was evaluated via the AllergenFP server (https://ddg-pharmfac.net/AllergenFP/ (accessed on 29 May 2025) Candidate epitopes identified as non-toxic and non-allergenic were selected for further analysis [27,28].

### 2.11. Molecular Docking

Molecular docking is a computational technique that predicts receptor-ligand interactions by minimizing the binding energy between three-dimensional protein structures. The amino acid sequences of Omp16 and Omp19 were retrieved from the UniProt database, while the crystal structures of the receptor proteins used for docking were obtained from the RCSB PDB database (https://www.rcsb.org/ (accessed on 5 June 2025) [29]. The most prevalent MHC class I and II alleles in the Chinese population, HLA-A*02:01 and HLA-DRB1*15:01, were selected to form complexes with their corresponding T-cell receptors (TCRs). Molecular docking was performed between Omp16 (ID: P0A3S7) and Omp19 (ID: P0A3P1) and the following receptors: ① B-cell receptor (BCR, PDB ID: 5IFH); ② HLA-A*02:01 (PDB ID: 6TDS) complexed with a cytotoxic T lymphocyte TCR (PDB ID: 5YXN); and ③ HLA-DRB1*15:01 (PDB ID: 8TBP) complexed with a helper T lymphocyte TCR (PDB ID: 1YMM). Docking simulations were carried out using AutoDock Vina 1.1.2. Prior to docking, all receptor structures were preprocessed with PyMol 2.5.2. The results were visualized and analyzed to evaluate the binding affinity of Omp16 and Omp19 to immune receptors and MHC molecules.

### 2.12. Immune Simulation

Immune stimulation simulations were performed using the C-ImmSim server (https://150.146.2.1/C-IMMSIM/index.php (accessed on 14 June 2025) to predict the expression dynamics of T cells, B cells, and cytokines in response to Omp16 and Omp19 protein stimulation [30].Parameter configurations were set as follows: random seed and simulation volume were set to default values; HLA alleles were selected according to the server’s recommended settings. The simulation was run for 800-time steps. Three injections of Omp16 protein were administered at days 0, 28, and 42, respectively.

## 3. Results

### 3.1. Constructing Recombinant Plasmids and Synthesizing mRNA via in Vitro Transcription

**Figure 1:**
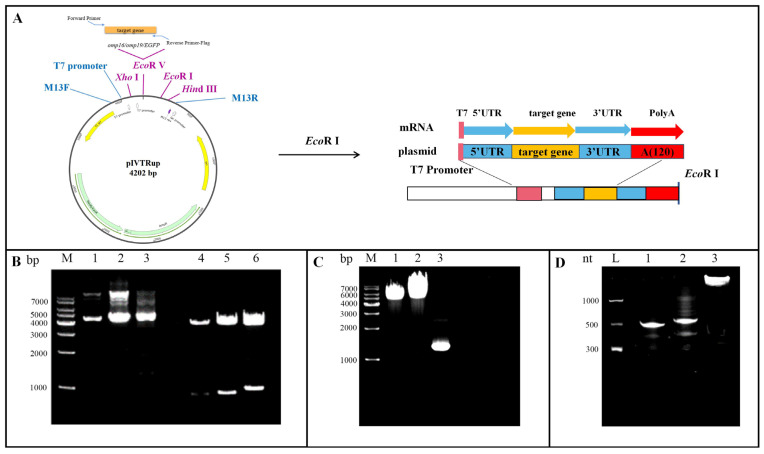
Construction of the recombinant plasmids pIVTRup-*omp16*, pIVTRup-*omp19*, and pIVTRup-*EGFP*. (**A**) Schematic diagram of plasmid construction and in vitro transcription of mRNA. (**B**) Identification of recombinant plasmids by double restriction enzyme digestion (m: 100 bp DNA marker; lanes 1–3: recombinant plasmids pIVTRup-*omp16*, pIVTRup-*omp19*, and pIVTRup-*EGFP*, respectively; lanes 4–6: products after digestion with *Xho* I and *Hind* III). (**C**) Preparation of DNA templates for in vitro transcription (M: 1 kb DNA marker; lane 1: linearized plasmid pIVTRup-*omp16*; lane 2: linearized plasmid pIVTRup-*omp19*; lane 3: PCR product of plasmid pIVTRup-*EGFP*). (**D**) Denaturing PAGE electrophoresis of purified mRNA (L: RNA Ladder; lane 1: *omp16*-mRNA; lane 2: *omp19*-mRNA; lane 3: *EGFP*-mRNA).

The target genes *omp16* (about 507 bp), *omp19* (about 534 bp), and *EGFP* (about 1082 bp) were amplified by PCR. The resulting fragments were ligated into a vector and transformed into *E. coli* competent cells. Positive clones were screened and identified by double digestion with *Xho* I and *Hind* III. The results showed that the digested plasmid produced a band at 4200 bp, while the target genes appeared as bands below 1000 bp (Figure 1B). Sequencing of the recombinant plasmids and alignment with reference sequences from GenBank confirmed that the *omp16* and *omp19* gene sequences were 100% correct. The recombinant plasmids pIVTRup-*omp16*/*omp19* were linearized by digestion with *Eco*R I, and the full-length *EGFP* functional region was obtained by amplification. As shown in Figure 1C, the product sizes were consistent with expectations. Using an in vitro transcription kit, linearized recombinant plasmids and the amplified *EGFP* product were used as templates to synthesize structurally intact and mature *omp16*-mRNA, *omp19*-mRNA, and *EGFP*-mRNA. Electrophoresis on a 5% denaturing PAGE gel revealed that the *omp16*-mRNA and *omp19*-mRNA were approximately 600 nt in length, and the *EGFP*-mRNA was approximately 1200 nt, which matched the predicted sizes (Figure 1D).

### 3.2. In Vitro Cell Experiments

**Figure 2:**
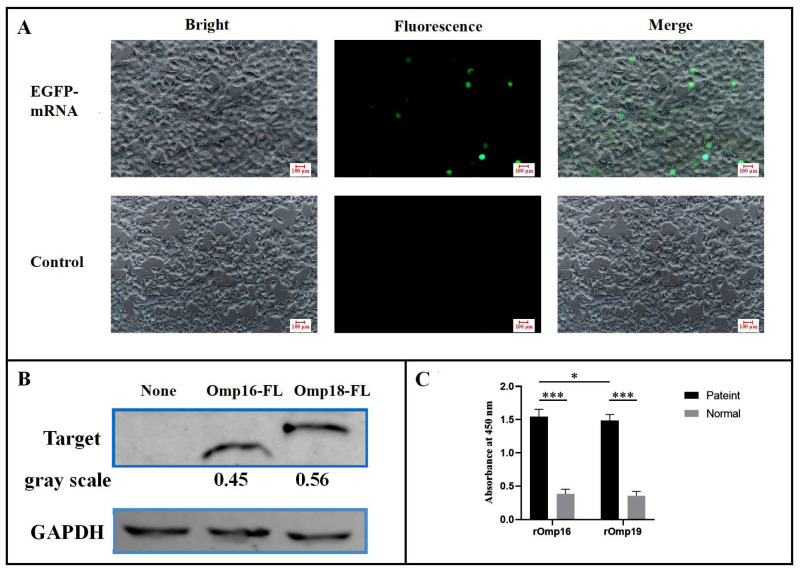
Detection of protein expression and immunogenicity in mRNA-transfected cells. (**A**) Fluorescence and bright-field images of HEK 293T cells 24 h after transfection with EGFP-mRNA or untreated control. (**B**) Western blot analysis of protein expression in mRNA-transfected cells. Proteins were detected using an anti-Flag primary antibody and a fluorescently labeled goat anti-mouse IgG secondary antibody; GAPDH was used as the loading control. Lanes 1, 2, and 3 correspond to the *EGFP*-mRNA, *omp16*-mRNA, and *omp19*-mRNA transfection groups, respectively. (**C**) Evaluation of the immunogenicity of rOmp16 and rOmp19 by indirect ELISA. Results are presented as mean OD_450_ values ± standard deviation after reaction with patient sera. Both rOmp16 and rOmp19 showed specific binding to antibodies in brucellosis patient sera compared to healthy control serum (*** *p* < 0.001). The binding affinity of rOmp16 was significantly stronger than that of rOmp19 (* *p* < 0.05).

Twenty-four hours after transfection of HEK 293T cells with in vitro transcribed mRNA, green fluorescent protein expression was observed under fluorescence microscopy in the *EGFP*-mRNA group, while no fluorescence was detected in the blank control group (Figure 2A). The transfection efficiency was estimated to be approximately 14.1% using ImageJ 1.54g. Using the *EGFP*-mRNA group as a reference, these results indicate that the mRNA was successfully translated into functionally intact protein in HEK 293T cells. The target proteins were purified using affinity resin conjugated with anti-Flag antibodies. Western blot analysis revealed protein bands at approximately 16 kDa for the *omp16*-mRNA group and 19 kDa for the *omp19*-mRNA group, consistent with expected sizes, while no obvious band was observed in the *EGFP*-mRNA group (Figure 2B). These findings demonstrate that both *omp16*-mRNA and *omp19*-mRNA were stably expressed in HEK 293T cells. To further investigate the immunogenicity of the expressed proteins, an indirect ELISA was performed. Wells were coated with 0.6 μg of purified recombinant protein antigen per well and incubated with diluted serum from patients with brucellosis, using healthy human serum as a control. The results showed that both rOmp16 and rOmp19 proteins specifically bound to antibodies in the patient sera (*** *p* <0.001), and the binding affinity of rOmp16 was significantly stronger than that of rOmp19 (* *p* <0.05) (Figure 2C).

### 3.3. Prediction of Antigenic Epitopes in Omp16 and Omp19 Proteins

#### 3.3.1. Prediction of B-Cell Antigenic Epitopes in Omp16 and Omp19 Proteins

Based on hidden Markov models, artificial neural networks, and support vector machine algorithms, linear B-cell epitopes of Omp16 and Omp19 proteins were predicted using the online tools BepiPred 2.0, ABCPred, and SVMTriP. High-scoring sequences were identified (Table 3 and Table 4). Specifically, the IEDB BepiPred 2.0 database was employed to predict linear B-cell epitopes using a response threshold of 0.5. Ultimately, overlapping sequences predicted by multiple tools were selected as final linear B-cell epitopes. For Omp16, the chosen epitopes were L_27–38_ (SKKNLPNNAGDL), L_62–72_ (FFDLDSSLIRA) and L_100–110_ (DERGTREYNLA). For Omp19, the selected epitopes included L_74–89_ (TQVASLPPASAPDLTP), L_111–121_ (QTKYGQGYRAG) and L_160–171_ (QGRFDGQTTGGQ) (Table 5).

#### 3.3.2. Prediction of T-Cell Antigenic Epitopes in Omp16 and Omp19 Proteins

Cytotoxic T lymphocyte (CTL) epitopes for Omp16 and Omp19 proteins were predicted using the IEDB (MHC-I Binding, TAP Transport, and MHC-I Immunogenicity) and SYFPEITHI databases. High-scoring overlapping sequences were selected as candidate epitopes (Table 6 and Table 7). The final selected CTL epitopes for Omp16 were L_9–17_ (RSPIAIALF), L_21–29_ (AVAGCASKK), L_87–95_ (YPQYSITI) and L_118–126_ (ATRDFLASR). For Omp19, the CTL epitopes chosen were L_10–19_ (SLAAAGIVL), L_99–107_ (SLGGQSCKI) and L_136–144_ (AVNGKQLVL) (Table 5). Helper T lymphocyte (HTL) epitopes were predicted using the IEDB (MHC-II Binding) and SYFPEITHI databases, with high-scoring overlapping sequences identified as candidate epitopes (Table 8 and Table 9). The final HTL epitopes selected for Omp16 included L_11–25_ (PIAIALFMSLAVAGC), L_58–72_ (GDRIFFDLDSSLIRA) and L_119–132_ (TRDFLASRGVPTN). For Omp19, the HTL epitopes chosen were L_57–71_ (PTQFPNAPSTDMSAQ) and L_140–151_ (KQLVLYDANGGTVAS) (Table 5).

#### 3.3.3. Prediction of Allergenicity and Toxicity

Prior to the consideration of viable candidate vaccines, it is essential to analyze the allergenicity and toxicity profiles of the conserved epitopes identified in the previous stages. Subsequent evaluation using AllergenFP and ToxinPred confirmed the selection of non-allergenic and non-toxic epitopes (Table 10). For the Omp16 protein, the following epitopes were selected: two B-cell epitopes: L_62–72_ (FFDLDSSLIRA) and L_100–110_ (DERGTREYNLA); two CTL epitopes: L_9–17_ (RSPIAIALF) and L_118–126_ (ATRDFLASR); two HTL epitopes: L_11–25_ (PIAIALFMSLAVAGC) and L_58–72_ (GDRIFFDLDSSLIRA). For the Omp19 protein, the selected epitopes included: three B-cell epitopes: L_74–89_ (TQVASLPPASAPDLTP), L_111–121_ (QTKYGQGYRAG) and L_160–171_ (QGRFDGQTTGGQ); one CTL epitope: L_136–144_ (AVNGKQLVL); two HTL epitopes: L_57–71_ (PTQFPNAPSTDMSAQ) and L_140–151_ (KQLVLYDANGGTVAS).

### 3.4. Molecular Docking

**Figure 3:**
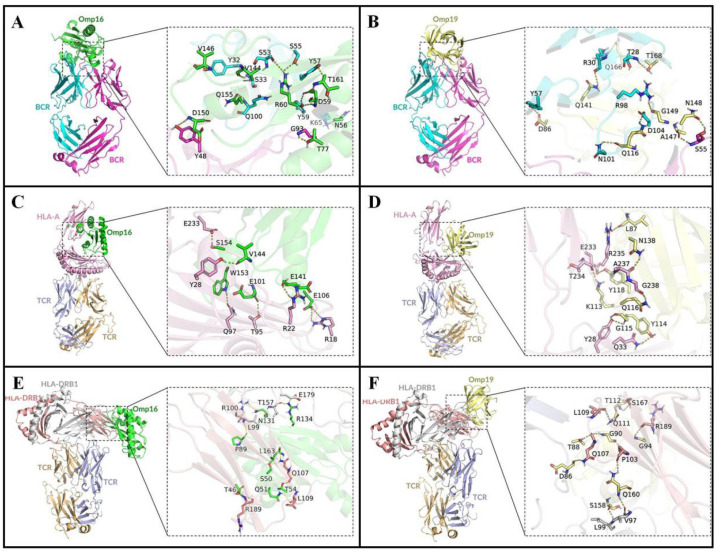
Molecular docking of antigenic proteins with target receptors. (**A**) Molecular docking of Omp16 protein with BCR. (**B**) Molecular docking of Omp19 protein with BCR. (**C**) Docking of Omp16 with the HLA-A*02:01–TCR complex. (**D**) Docking of Omp19 with the HLA-A*02:01–TCR complex. (**E**) Docking of Omp16 with the HLA-DRB1*15:01–TCR complex. (**F**) Docking of Omp19 with the HLA-DRB1*15:01–TCR complex. Hydrogen bonds are indicated by dashed lines.

The docking simulations revealed that both Omp16 and Omp19 form stable complexes with their respective target receptors (Figure 3). Specifically, the interaction between Omp16 and BCR involved 11 hydrogen bonds, while Omp19 bound to BCR through 9 hydrogen bonds (Figure 3A,B). In the ternary complexes with HLA-A*02:01 and the CTL-surface TCR, Omp16 engaged in 8 hydrogen bonds, compared to 6 for Omp19 (Figure 3C,D). Both proteins formed interactions involving 9 hydrogen bonds within the complexes comprising HLA-DRB1*15:01 and the HTL-surface TCR (Figure 3E,F). These results indicate that Omp16 exhibits a superior binding stability relative to Omp19 in complexes with both BCR and MHC class I–TCR, suggesting its enhanced potential in activating humoral immune responses and cytotoxic T-cell mechanisms.

### 3.5. Immune Simulation

#### 3.5.1. Immune Stimulation of Innate Immune Cells by Omp16 and Omp19 Proteins

**Figure 4:**
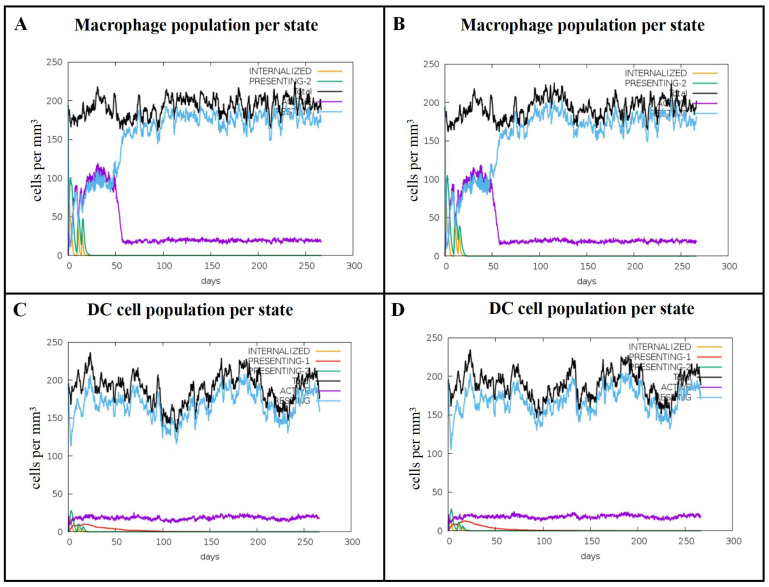
Predicted immune stimulation of innate immune cells. (**A**) Macrophage counts following Omp16 injection. (**B**) Macrophage counts following Omp19 injection. (**C**) DC counts following Omp16 injection. (**D**) DC counts following Omp19 injection.

The C-ImmSim server is a computational tool designed for simulating immune responses. Simulations performed using this platform allow for the observation of dynamic changes in human immune cells. Results from C-ImmSim indicated that both Omp16 and Omp19 proteins significantly activated macrophages (Figure 4A,B). Specifically, the population of active macrophages remained at approximately 150 cells/mm^3^ from the first to the third immunization, followed by a rapid decline to around 20 cells/mm^3^ by 60 days after the initial inoculation. Furthermore, dendritic cells (DCs), as the most potent antigen-presenting cells (APCs), play a critical role in presenting vaccine antigens to T cells. Our simulation showed that vaccination with Omp16 and Omp19 also induced significant activation of DCs (Figure 4C,D). Upon protein stimulation, the total number of DCs per state stabilized at approximately 100 cells/mm^3^, while the count of activated DCs increased rapidly at a rate of 20 cells/mm^3^ per state.

#### 3.5.2. Immune Stimulation of Adaptive Immune Cells by Omp16 and Omp19 Proteins

**Figure 5:**
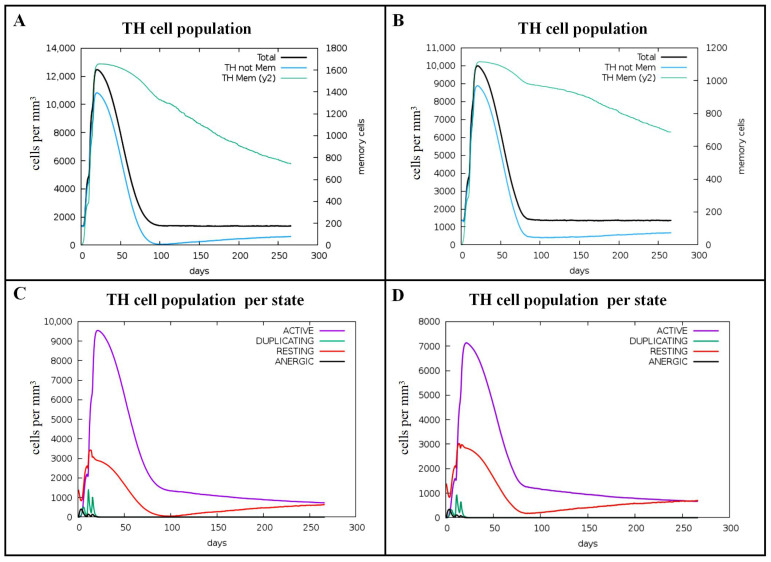
Predicted immune stimulation of CD4^+^ T cells. (**A**) TH cell counts after Omp16 injection. (**B**) TH cell counts after Omp19 injection. (**C**) TH cell counts by state after Omp16 injection. (**D**) TH cell counts by state after Omp19 injection. CTLs contribute to the clearance of *Brucella* through the production of perforin, granzyme B, and other cytotoxic factors.

Adaptive immune cells, primarily including CD4^+^ T cells, CD8^+^ T cells, and B cells, play a critical role in counteracting the pathogenesis of *Brucella*. Our results demonstrated that stimulation with Omp16 protein induced a significant increase in the populations of total T helper (TH) cells, non-memory TH cells, and memory TH cells, which peaked after the second stimulation (Figure 5A, reaching approximately 12,500 cells/mm^3^, 11,000 cells/mm^3^, and 1600 cells/mm^3^, respectively). Concurrently, the numbers of active and resting CD4^+^ T cells per state also reached their highest levels post-second stimulation, at 9500 cells/mm^3^ and 3500 cells/mm^3^, respectively (Figure 5C). In contrast, Omp19 protein stimulation resulted in a comparatively lower increase in CD4^+^ T cell quantities. These also peaked following the second stimulation, with total TH cells, non-memory TH cells, and memory TH cells reaching approximately 10,000 cells/mm^3^, 9000 cells/mm^3^, and 1100 cells/mm^3^, respectively (Figure 5B). After the second stimulation, the counts of active and resting CD4^+^ T cells per state peaked at 7000 cells/mm^3^ and 3000 cells/mm^3^, respectively (Figure 5D).

**Figure 6:**
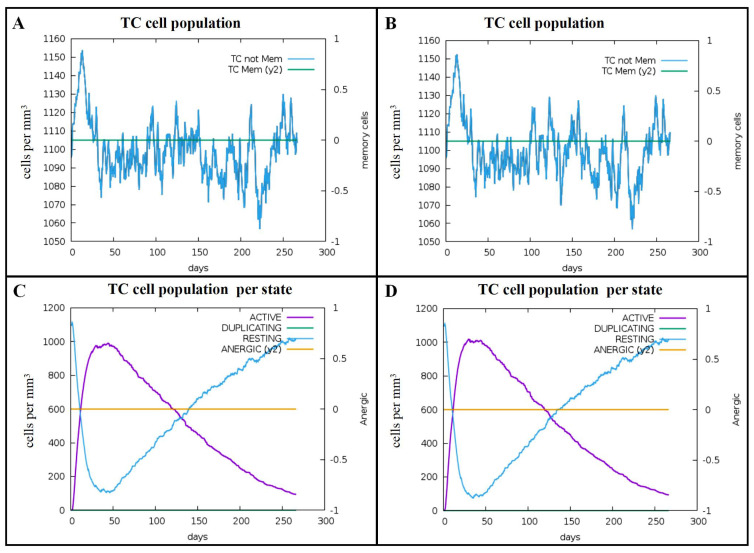
Predicted immune stimulation of CD8^+^ T cells. (**A**) TC cell counts after Omp16 injection. (**B**) TC cell counts after Omp19 injection. (**C**) TC cell counts by state after Omp16 injection. (**D**) TC cell counts by state after Omp19 injection.

According to the C-ImmSim server results, two stimulations with Omp16 and Omp19 proteins induced a peak in the number of non-memory CTLs per state, reaching maximum values of 1150 cells/mm^3^ and 1155 cells/mm^3^, respectively (Figure 6A,B). Furthermore, around day 40 after the initial immunization, the population of active CTLs per state peaked at 1000 cells/mm^3^ for both proteins, while the number of inactive CTLs per state reached a trough of 100 cells/mm^3^ (Figure 6C,D).

**Figure 7:**
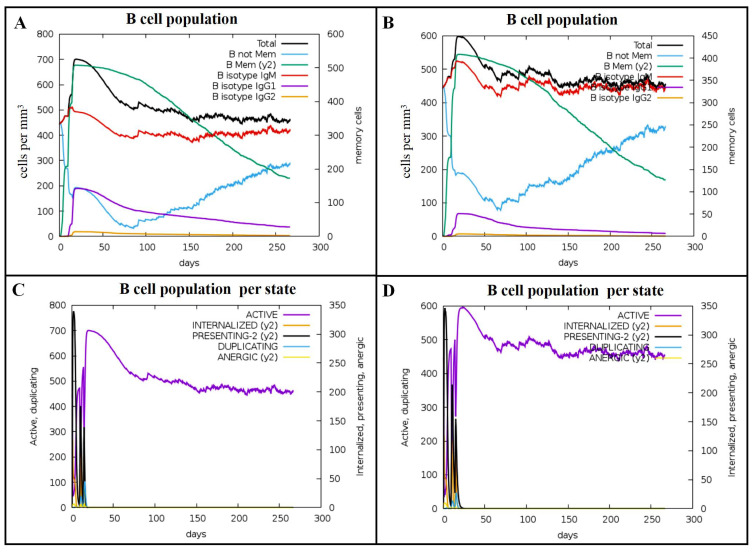
Predicted immune stimulation of B cells. (**A**) B cell counts after Omp16 injection. (**B**) B cell counts after Omp19 injection. (**C**) B cell counts by state after Omp16 injection. (**D**) B cell counts by state after Omp19 injection.

We also predicted the B-cell population per state following immunization with Omp16 and Omp19 proteins. The results indicated that after the second stimulation with Omp16, the total B-cell count peaked at 700 cells/mm^3^ (Figure 7A). The number of active B cells reached a maximum of 700 cells/mm^3^ post-second stimulation, while preseting-2 cells peaked at 340 cells/mm^3^ after the first stimulation (Figure 7C). In contrast, immunization with Omp19 elicited a slightly lower B-cell response. The total B-cell count reached a peak of 600 cells/mm^3^ following the second stimulation (Figure 7B). Active B cells peaked at 600 cells/mm^3^ after the second stimulation, and preseting-2 cells reached a maximum of 350 cells/mm^3^ after the first stimulation (Figure 7D).

#### 3.5.3. Humoral Immune Response Induced by Omp16 and Omp19 Proteins

**Figure 8:**
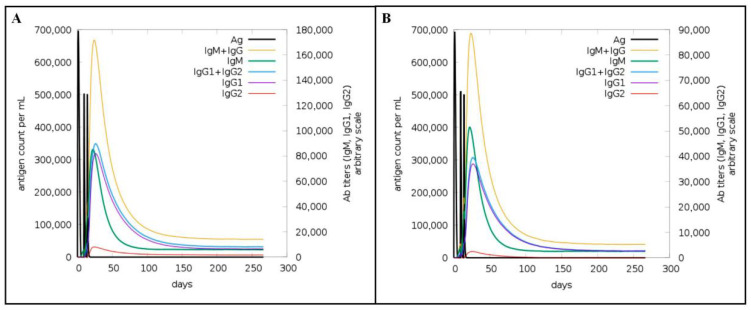
Predicted immune stimulation of B cells. (**A**) Antibody level after Omp16 injection. (**B**) Antibody level after Omp19 injection.

In computer analysis, IgM antibody levels peaked at 85,000/mL and 50,000/mL, respectively, after the second injection of Omp16 and Omp19 proteins, and then gradually decreased (Figure 8). IgG1 + IgG2 antibody levels peaked at 350,000/mL and 300,000/mL, respectively, after the second immunization, and then gradually decreased. Therefore, Omp16 was able to produce higher levels of specific antibodies.

## 4. Discussion

*Brucella*, as an intracellular parasite, survives and replicates in macrophages and placental trophoblasts by disrupting the host immune response through immune evasion mechanisms [31,32]. Upon injection, mRNA vaccines encapsulated in lipid nanoparticles (LNPs) are taken up by activated monocytes and DCs. These cells synthesize the encoded antigenic proteins and present the processed antigens in lymphoid tissues, effectively driving adaptive immune responses and demonstrating strong efficacy in activating cellular immunity [33].

The selection of appropriate antigens is crucial for the development of mRNA vaccines. Omp16 and Omp19 are outer membrane lipoproteins that act as pathogen-associated molecular patterns of *Brucella* and can activate DCs and induce Th1 immune responses in vivo [34]. Extensive research has been conducted on DNA vaccines and recombinant protein vaccines targeting Omp16 and Omp19. The molecular weights of rOmp16 and rOmp19 proteins expressed and purified by prokaryotic expression are 16 kDa and 19 kDa respectively, which is consistent with Western blot results [35]. *EGFP*-mRNA was prepared with *EGFP* as reporter gene, and green fluorescence was observed 24 h after transfection into HEK 293T cells, which further confirmed the successful expression of EGFP-mRNA in eukaryotic expression system.

Most eukaryotic mRNAs require a 7-methylguanosine at the 5 ‘end We synthesized the complete mRNA using the ARCA cap analog in the in vitro transcription kit and the Poly (A) tail sequence of the template plasmid, but it can only be used for in vitro cell transfection experiments. For in vivo immunogenicity studies in animals, the incorporation of signal peptide sequences at the 5′ end is essential. This modification enables the translated antigen to be directed through the endoplasmic reticulum/Golgi pathway, facilitating its secretion into the extracellular space [36]. There are two major obstacles in the development of mRNA vaccines: mRNA instability and high inflammatory response [37,38]. Using ionizable lipid nanoparticle technology, intact mRNA can be efficiently delivered into the cytoplasm of cells and then translated into encoded antigenic proteins [39]. Dogo 4 is a less toxic, mannose functionalized liposome that efficiently delivers siRNA in vitro and in vivo [40]. In this study, Dogo 4 liposomes were used to encapsulate and transport mRNA into cells, greatly preserving mRNA integrity and improving transfection efficiency.

The purified rOmp16 and rOmp19 proteins were reacted with serum samples from patients infected with *Brucella*. Analysis revealed that both proteins specifically bound to IgG antibodies in the patient sera, indicating that the in vitro transcribed mRNA was successfully expressed in the eukaryotic system and retained immunogenicity. This finding indirectly validates the initial step of mRNA vaccine-induced immune activation in vivo. However, further animal studies are required to assess the in vivo expression efficiency of the mRNA and its ability to induce antibody production. Notably, we observed differential reactivity between rOmp16 and rOmp19 proteins expressed in mRNA-transfected cells when probed with patient sera, while no such difference was detected in the healthy control group. This discrepancy is particularly intriguing and warrants deeper exploration beyond simply attributing it to variations in immunogenicity. The stronger binding affinity of rOmp16 compared to rOmp19 (as shown in Figure 2C) suggests that rOmp16 may possess a higher density of accessible epitopes, or perhaps its epitopes are more conformationally stable and better recognized by the circulating antibodies in brucellosis patients. This could be due to structural differences between the two proteins, leading to varying degrees of antigen processing and presentation in vivo, or differential exposure of key antigenic determinants during natural infection. Future studies could involve detailed structural analysis of rOmp16 and rOmp19, as well as epitope mapping using patient sera, to elucidate the molecular basis for this observed difference in immunogenicity. Understanding these nuances is critical for optimizing antigen selection and design in multi-epitope mRNA vaccines.

Furthermore, this study predicted the dominant epitopes of Omp16 and Omp19 proteins and performed molecular docking with MHC molecules and surface receptors of immune cells to investigate their mechanisms of specific binding to antibodies in patient sera, as well as their ability to elicit humoral and cell-mediated immune responses. B-cell epitope prediction tools utilize the principle that complementarity-determining regions (CDRs) of antibodies recognize antigenic epitopes with high specificity. By analyzing antibody–antigen protein structures, these tools enable accurate prediction of epitopes across diverse antigens [41]. B-cell epitopes of Omp16 and Omp19 were systematically mapped with the ABCpred algorithm, mirroring the epitope-prediction workflow previously established for the L7/L12 ribosomal protein [25]. Two dominant epitopes were identified in Omp16 (L_62–72_ and L_100–110_), and three were identified in Omp19 (L_74–89_, L_111–121_, and L_160–171_). In contrast, predictions of CTL and HTL epitopes were based on the binding affinity between antigenic peptides and MHC class I and II molecules [42,43]. Through integrated use of IEDB and SYFPEITHI, we identified two CTL epitopes (L_9–17_ and L_118–126_) and two HTL epitopes (L_11–25_ and L_58–72_) for Omp16, while one CTL epitope (L_136–144_) and two HTL epitopes (L_57–71_ and L_140–151_) were predicted for Omp19. These dominant epitopes critically determine the immunogenicity of the antigens and play essential roles in the immune response against *Brucella* infection. Multi-epitope vaccine, constructed by linking and optimizing these epitopes, have been shown to induce high levels of both humoral and cellular immunity in mice [44,45]. The identification of these specific B-cell, CTL, and HTL epitopes provides a rational basis for the design of a multi-epitope mRNA vaccine. By focusing on these immunodominant regions, we can potentially enhance the breadth and magnitude of the immune response, leading to more effective protection. For instance, the presence of multiple CTL epitopes suggests that both Omp16 and Omp19 can effectively stimulate cytotoxic T-lymphocyte responses, which are crucial for clearing intracellular pathogens like *Brucella*. The distinct sets of epitopes identified for Omp16 and Omp19 also highlight the potential benefit of combining both antigens in a vaccine strategy to achieve a more comprehensive immune coverage against different strains or stages of infection.

In the immune response to *Brucella* infection, bacterial antigens are phagocytized by antigen presenting cells and processed into antigenic epitopes. These peptide fragments are present on the cell surface and can be recognized and bound by immune cells. Docking results of Omp16 and Omp19 with BCR, TCR and MHC molecules show that Omp16 and Omp19 can form stable complexes with them and form a large number of hydrogen bonds. Hydrogen bonds reduce the energy of the complex system and the binding tends to stabilize [46]. For example, aspartic acid in L_74–89_ and glycine in L_112–122_ can form hydrogen bonds with BCR, and asparagine in L_136–144_ can form hydrogen bonds with MHCI-TCR complexes. The molecular docking analysis provides crucial insights into the structural basis of antigen-receptor interactions. The observation that Omp16 forms more hydrogen bonds with BCR and MHC class I-TCR complexes compared to Omp19 (11 vs. 9 with BCR; 8 vs. 6 with MHC I-TCR) is significant. This suggests a potentially stronger and more stable binding affinity for Omp16 with these key immune receptors. A higher number of hydrogen bonds implies a more robust interaction, which could translate to more efficient antigen presentation and subsequent activation of B cells and cytotoxic T cells. This finding aligns with our ELISA results showing stronger immunogenicity for rOmp16 and provides a mechanistic explanation at the molecular level. For vaccine design, this suggests that Omp16 might be a more potent immunogen, or that specific modifications to Omp19 could be explored to enhance its binding stability and immunogenicity. Further computational and experimental validation of these binding energies and their correlation with immune activation would be valuable.

To further characterize the immunoinformatic profiles of Omp16 and Omp19, we utilized the C-IMMsim server to simulate cell-mediated immune responses induced by an MP3RT vaccine formulation. The simulation results demonstrated that following three successive immunizations, the number of activated macrophages remained stable at approximately 100 cells/mm^3^, while the total DCs count was maintained at around 150 cells/mm^3^, with actively secreting DCs stabilizing at 20 cells/mm^3^. These data suggest that both Omp16 and Omp19 can initiate innate immune responses by activating macrophages and DCs. The C-ImmSim results are highly encouraging, as they predict a robust activation of innate immune cells (macrophages and DCs) by both Omp16 and Omp19. The sustained levels of activated macrophages and DCs after multiple immunizations indicate that these antigens can effectively prime the immune system for adaptive responses. DCs, as professional antigen-presenting cells, are critical for initiating T cell responses. Their significant activation suggests that the mRNA vaccine encoding Omp16 and Omp19 would efficiently present antigens to T cells, thereby bridging innate and adaptive immunity. The stability of these cell populations over time also implies a sustained immune surveillance, which is desirable for long-term protection against *Brucella*.

Regarding adaptive immune responses, the simulation showed that Omp16 stimulation led to a higher peak in total T helper (TH) cells, non-memory TH cells, and memory TH cells compared to Omp19. Specifically, Omp16 induced approximately 12,500 cells/mm^3^ total TH cells, while Omp19 induced around 10,000 cells/mm^3^. This difference in TH cell activation is crucial because CD4^+^ TH cells play a central role in coordinating both humoral and cellular immunity. A stronger TH cell response, particularly the generation of memory TH cells, suggests a more potent and long-lasting adaptive immune response. Similarly, while both proteins induced comparable peaks in non-memory cytotoxic CTLs, the overall kinetics and magnitude of the CD4^+^ T cell response appear more favorable with Omp16. This reinforces the notion from ELISA and molecular docking that Omp16 might be a more potent component for inducing a comprehensive adaptive immune response. The predicted B-cell responses also showed Omp16 inducing a slightly higher total B-cell count and active B cells compared to Omp19 (700 vs. 600 cells/mm^3^). This further supports the stronger humoral response observed with Omp16. The higher IgM and IgG1 + IgG2 antibody levels predicted for Omp16 (85,000/mL IgM and 350,000/mL IgG for Omp16 vs. 50,000/mL IgM and 300,000/mL IgG for Omp19) directly correlate with its superior ability to elicit specific antibodies, which is a cornerstone of protective immunity against extracellular pathogens and toxins, and also contributes to intracellular pathogen clearance.

While these in silico modeling results provide valuable insights into the potential immune responses elicited by Omp16 and Omp19, it is crucial to acknowledge that computational predictions are not a substitute for experimental validation. The C-ImmSim server offers a powerful tool for hypothesis generation and preliminary assessment of immunogenicity profiles; however, the complex interplay of immune cells and molecules in a living organism cannot be fully replicated in a simulated environment. Therefore, the next critical steps in the development of this mRNA vaccine candidate must involve rigorous in vivo studies. These will include evaluating the actual immunogenicity of the mRNA vaccine in animal models (e.g., mice or guinea pigs), assessing the magnitude and quality of both humoral and cellular immune responses, and most importantly, conducting challenge studies to determine the protective efficacy against B. melitensis infection. Therefore, we further evaluated the immunogenicity by predicting epitopes for common mouse MHC alleles (See Supplementary Materials Tables S1–S5). Such in vivo experiments are indispensable for confirming the translational potential of our in silico and in vitro findings and moving closer to a viable human vaccine.

In summary, the integrated bioinformatic and in vitro experimental findings strongly support the potential of Omp16 and Omp19 as promising antigens for a *B. melitensis* mRNA vaccine. The observed differences in immunogenicity and receptor binding between Omp16 and Omp19 highlight the importance of careful antigen selection and potentially the synergistic benefits of a multi-antigen vaccine approach. While this study provides a preliminary exploration, the successful expression of immunogenic proteins in eukaryotic cells, coupled with robust immune simulation predictions, lays a solid theoretical foundation. Future research should focus on in vivo animal studies to validate these findings, assess the protective efficacy of the mRNA vaccine, and further optimize the vaccine formulation and delivery system. Additionally, investigating the precise mechanisms underlying the differential immunogenicity of Omp16 and Omp19 at a structural and cellular level will be crucial for the rational design of next-generation brucellosis vaccines.

## 5. Conclusions

In this study, mRNA sequences encoding the *omp16* and *omp19* genes of *B. melitensis* were synthesized via in vitro transcription. The resulting mRNAs mediated the expression of immunogenic proteins in mammalian cells. Furthermore, bioinformatic approaches were employed to identify dominant antigenic epitopes of Omp16 and Omp19 and to simulate immune response profiles following vaccination. These findings provide a foundational investigation for the development of an mRNA-based vaccine against *B. melitensis*.

## Figures and Tables

**Table 1:** Primers of PCR for target gene ^&^.

Primer Name	Primer Sequence (5′-3′)
OMP16-F	cccaagcttatgcgccgtatccagtcg
OMP16-R-FLAG	cgcggatccttacttgtcatcgtcgtccttgtagtcccgtccggccccgttgag
OMP19-F	cccaagcttatgggaatttcaaaagcaag
OMP19-R-FLAG	cgcggatccttacttgtcatcgtcgtccttgtagtcgcgcgacagcgtcacggcc
EGFP-F	cgcaaatgggcggtaggcgtg
EGFP-R	atttaggtgacactatag
M13R	caggaaacagctatgacc

^&^ Note: The underlined part is the Flag tag sequence.

**Table 2:** In vitro transcription system.

Reagent	Dosage
2 × ARCA/NTP Mix	5 µL
DDT (0.1 M)	0.5 µL
Template DNA	500 ng
T7 RNA Polymerase Mix	1 µL
ddH_2_O	Add to10 μL

**Table 3:** Prediction of B-cell epitopes in Omp16 protein ^&^.

Method	Position	Sequence	Score
Bepi Pred	27	SKKNLPNNAGDLGLG	0.6
	62	FFDLDSSLIRA	0.6
	100	DERGTREYNLA	0.5
	136	ISYGNERPVAVCDADTCWSQ	0.5
ABC Pred	38	LGLGAGAATPGSSQDF	0.9
	96	EGHADERGTREYNLAL	0.9
	23	AGCASKKNLPNNAGDL	0.8
	133	MRTISYGNERPVAVCD	0.8
SVMTrip	60	RIFFDLDSSLIRAD	1.0

^&^ Note: The underlined part is the overlapping segments detected by three methods: BepiPred, ABCPred, and SVMTrip.

**Table 4:** Prediction of B-cell epitopes in Omp19 protein ^&^.

Method	Position	Sequence	Score
Bepi Pred	23	SSRLGNLDNVSPP	0.6
	68	DMSAQSGTQVA	0.6
	111	QTKYGQGYRAGPLRCPGELANLASWAV	0.5
	157	SSGQGRFDGQTTGGQA	0.5
ABC Pred	74	TQVASLPPASAPDLTP	0.9
	107	IATPQTKYGQGYRAGP	0.9
	82	ASAPDLTPGAVAGVWN	0.9
	160	QGRFDGQTTGGQAVTL	0.8
SVMTrip	132	LASWAVNGKQLVLY	1.0

^&^ Note: The underlined part is the overlapping segments detected by three methods: BepiPred, ABCPred, and SVMTrip.

**Table 5:** Dominant linear B-cell, CTL, and HTL epitopes of Omp16 and Omp19 proteins ^&^.

		Omp16		Omp19	
Epitope	Method	Position	Sequence	Position	Sequence
B cell	ABCPred, BepiPred 2.0, SVMTrip	27	SKKNLPNNAGDL	74	TQVASLPPASAPDLTP
		62	FFDLDSSLIRA	111	QTKYGQGYRAG
		100	DERGTREYNLA	160	QGRFDGQTTGGQ
CTL	IEDB-MHC I, SYFPEITHI	9	RSPIAIALF	10	SLAAAGIVL
		21	AVAGCASKK	99	SLGGQSCKI
		87	RYPQYSITI	136	AVNGKQLVL
		118	ATRDFLASR		
HTL	IEDB-MHC II, SYFPEITHI	11	PIAIALFMSLAVAGC	57	PTQFPNAPSTDMSAQ
		58	GDRIFFDLDSSLIRA	140	KQLVLYDANGGT
		119	TRDFLASRGVPTN		

^&^ Note: CTL = Cytotoxic T lymphocyte; HTL = Helper T lymphocyte; The underlined part is the overlapping segments from Table 3 and Table 4.

**Table 6:** Prediction of CTL epitopes in Omp16.

Method	Allele	Position	Sequence	Score
IEDB	HLA-A*24:02	9	RSPIAIALF	0.32
	HLA-C*07:02	8	ARSPIAIAL	0.24
SYFPEITHI	HLA-A*02:01	15	ALFMSLAVA	23
		6	SIARSPIAI	21
	HLA-A*11:01	21	AVAGCASKK	26
		118	ATRDFLASR	24
		134	RTISYGNER	21
	HLA-A*24:02	87	RYPQYSITI	22

**Table 7:** Prediction of CTL epitopes in Omp19.

Method	Allele	Position	Sequence	Score
IEDB	HLA-A*02:01	10	SLAAAGIVLA	0.28
	HLA-A*11:01	132	LASWAVNGK	0.26
SYFPEITHI	HLA-A*02:01	87	LTPGAVAGV	25
		10	SLAAAGIVL	24
		99	SLGGQSCKI	24
		147	ANGGTVASL	22
		136	AVNGKQLVL	21
	HLA-A*11:01	43	AVPAGTVQK	28
		98	ASLGGQSCK	25

**Table 8:** Prediction of HTL epitopes in Omp16.

Method	Allele	Position	Sequence	Score
IEDB	HLA-DRB1*09:01	119	TRDFLASRGVPTN	0.19
SYFPEITHI	HLA-DRB1*02:01	67	SSLIRADAQQTLSKQ	36
		58	GDRIFFDLDSSLIRA	29
		52	DFTVNVGDRIFFDLD	24
		60	RIFFDLDSSLIRADA	21
	HLA-DRB1*07:01	88	YPQYSITIEGHADER	28
		59	DRIFFDLDSSLIRAD	26
		11	PIAIALFMSLAVAGC	24
	HLA-DRB1*15:01	11	PIAIALFMSLAVAGC	34
		82	AQWLQRYPQYSITIE	24

**Table 9:** Prediction of HTL epitopes in Omp19.

Method	Allele	Position	Sequence	Score
IEDB	HLA-DRB1*15:01	140	KQLVLYDANGGT	0.01
SYFPEITHI	HLA-DRB1*02:01	140	KQLVLYDANGGTVAS	28
		152	VASLYSSGQGRFDGQ	26
	HLA-DRB1*07:01	57	PTQFPNAPSTDMSAQ	32
		89	PGAVAGVWNASLGGQ	25
		15	GIVLAGCQSSRLGNL	24
		126	PGELANLASWAVNGK	24
		76	VASLPPASAPDLTPG	22
	HLA-DRB1*15:01	139	GKQLVLYDANGGTVA	28
		23	SSRLGNLDNVSPPPP	24

**Table 10:** Prediction of Allergenicity and Toxicity.

		Sequence	Allergenicity	Toxicity
Omp16	B-cell epitopes	FFDLDSSLIRA	Non-Allergen	Non-Toxin
		DERGTREYNLA	Non-Allergen	Non-Toxin
	CTL epitopes	RSPIAIALF	Non-Allergen	Non-Toxin
		ATRDFLASR	Non-Allergen	Non-Toxin
	HTL epitopes	PIAIALFMSLAVAGC	Non-Allergen	Non-Toxin
		GDRIFFDLDSSLIRA	Non-Allergen	Non-Toxin
		TRDFLASRGVPTN	Non-Allergen	Non-Toxin
Omp19	B-cell epitopes	TQVASLPPASAPDLTP	Non-Allergen	Non-Toxin
		QTKYGQGYRAG	Non-Allergen	Non-Toxin
		QGRFDGQTTGGQ	Non-Allergen	Non-Toxin
	CTL epitopes	AVNGKQLVL	Non-Allergen	Non-Toxin
	HTL epitopes	PTQFPNAPSTDMSAQ	Non-Allergen	Non-Toxin
		KQLVLYDANGGT	Non-Allergen	Non-Toxin

## Data Availability

The original contributions presented in this study are included in the article/Supplementary Material. Further inquiries can be directed to the corresponding authors.

## Supplementary Materials

The following supporting information can be downloaded at: https://www.mdpi.com/article/10.3390/vaccines14030240/s1, Table S1: Prediction of CTL epitopes in Omp16; Table S2: Prediction of CTL epitopes in Omp19; Table S3: Prediction of HTL epitopes in Omp16; Table S4: Prediction of HTL epitopes in Omp19; Table S5: Prediction of Allergenicity and Toxicity.

## Abbreviations

The following abbreviations are used in this manuscript:

*B. melitensis*	*Brucella melitensis*
Omp	Outer membrane protein
EGFP	Enhanced green fluorescent protein
MHC	Major histocompatibility complex
CTL	Cytotoxic T lymphocyte
HTL	Helper T lymphocyte
APCs	Antigen-presenting cells
DCs	Dendritic cells
TH	T helper
LNPs	Lipid nanoparticles
TLR2	Toll-like receptor 2
