# Supplementary material for: Preparation and Immunogenicity Prediction of Brucella melitensis mRNA Vaccine Candidate Based on omp16 and omp19 Genes
Source: Vaccines (Basel). 2026 Mar 5;14(3):240. doi: 10.3390/vaccines14030240 (PMC13030652; doi:10.3390/vaccines14030240)
Supplement: Supplementary file 1 [file vaccines-14-00240-s001.zip › vaccines-4150304-supplementary.pdf]

Supplementary Materials

Prediction of T-cell antigenic epitopes for common mice MHC alleles

CTL epitopes for Omp16 and Omp19 proteins were predicted using the IEDB (MHC-I Binding, TAP Transport, and MHC-I Immunogenicity) and SYFPEITHI databases. We selected common alleles in mice for prediction, including six alleles of MHC class I molecules: H-2-Db, H-2-Dd, H-2-Kb, H-2-Kk, H-2-Kd, and H-2-Ld. High-scoring overlapping sequences were selected as candidate epitopes (Table S1-2). The final selected CTL epitopes for Omp16 were L_{9-20} (RSPIAIALFMSL), $L_{115-123}$ (RAAATRDFL) and L_{63-70} (FDLDSSLI). For Omp19, the CTL epitopes chosen were L_{79-87} (LPPASAPDL), L_{92-100} (VAGVWNASL) and $L_{134-142}$ (SWAVNGKQL).

Table S1. Prediction of CTL epitopes in Omp16.

Method	Allele	Position	Sequence	Score
	H-2-Dd	9	RSPIAIALFMSL	0.32
IEDB	H-2-Ld	10	SPIAIALFM	0.31
	H-2-Db	115	RAAATRDFL	0.23
	H-2-Db	30	NLPNNAGDL	66.7
	H-2-Kb	13	AIALFMSL	71
SYFPEITHI	H-2-Kd	13	AIALFMSL	71
	H-2-Kk	63	FDLDSSLI	76.7
	H-2-Ld	9	RSPIAIALF	64.5

Table S2. Prediction of CTL epitopes in Omp19.

Method	Allele	Position	Sequence	Score
IEDB	H-2-Kb	92	VAGVWNASL	0.30
	H-2-Db	134	SWAVNGKQL	66.7
SYFPEITHI	H-2-Kk	145	YDANGGTV	66.7
	H-2-Ld	79	LPPASAPDL	77.4

HTL epitopes were predicted using SYFPEITHI databases, with high-scoring sequences identified as candidate epitopes (Table S3-4). We selected common alleles in mice for prediction, including four alleles of MHC class Ⅱ molecules: H2-I-Ad, H2-I-Ak, H2-I-Ed, and H2-I-Ek. The final HTL epitopes selected for Omp16 included L_{13-27} (AIALFMSLAVAGCAS), $L_{105-119}$ (REYNLALGQRRAAAT) and $L_{138-152}$ (YGNERPVAVCDADTC). For Omp19, the HTL epitopes chosen were L_{14-28}(AGIVLAGCQSSRLGN), L_{35-49}(PPPPAPVNAVPAGTV) and $L_{161-175}$ (GRFDGQTTGGQAVTL).

Table S3. Prediction of HTL epitopes in Omp16.

Method	Allele	Position	Sequence	Score
	H2-I-Ad	138	YGNERPVAVCDADTC	91.2
SYFPEITHI	H2-I-Ak	105	REYNLALGQRRAAAT	87.5
	H2-I-Ed,	104	TREYNLALGQRRAAA	82.4
	H2-I-Ek	13	AIALFMSLAVAGCAS	71.4

Table S4. Prediction of HTL epitopes in Omp19.

Method	Allele	Position	Sequence	Score
	H2-I-Ad	35	PPPPAPVNAVPAGTV	82.4
SYFPEITHI	H2-I-Ak	161	GRFDGQTTGGQAVTL	62.5
	H2-I-Ed	14	AGIVLAGCQSSRLGN	78.6
	H2-I-Ek	38	PAPVNAVPAGTVQKG	71.4

The toxicity and allergenicity of each epitope were assessed using the ToxinPred server and AllergenFP, respectively. Candidate epitopes identified as non-toxic and non-allergenic were selected for further analysis (Table S5). For the Omp16 protein, the selected epitopes included three CTL epitopes: L_{9-20} (RSPIAIALFMSL), $L_{115-123}$ (RAAATRDFL) and L_{63-70} (FDLDSSLI); and three HTL epitopes: L_{13-27} (AIALFMSLAVAGCAS), $L_{105-119}$ (REYNLALGQRRAAAT) and $L_{138-152}$ (YGNERPVAVCDADTC). For the Omp19 protein, the selected epitopes included two CTL epitopes: L_{79-87} (LPPASAPDL) and L_{92-100} (VAGVWNASL); and one HTL epitope: L_{35-49}(PPPPAPVNAVPAGTV).

Table S5. Prediction of Allergenicity and Toxicity.

		Sequence	Allergenicity	Toxicity
Omp16	CTL epitopes	RSPIAIALFMSL	Non-Allergen	Non-Toxin
		RAAATRDFL	Non-Allergen	Non-Toxin
		FDLDSSLI	Non-Allergen	Non-Toxin
	HTL epitopes	AIALFMSLAVAGCAS	Non-Allergen	Non-Toxin
		REYNLALGQRRAAAT	Non-Allergen	Non-Toxin
		YGNERPVAVCDADTC	Non-Allergen	Non-Toxin
Omp19	CTL epitopes	LPPASAPDL	Non-Allergen	Non-Toxin
		VAGVWNASL	Non-Allergen	Non-Toxin
		SWAVNGKQL	Allergen	Non-Toxin
	HTL epitopes	AGIVLAGCQSSRLGN	Allergen	Non-Toxin
		PPPPAPVNAVPAGTV	Non-Allergen	Non-Toxin
		GRFDGQTTGGQAVTL	Allergen	Non-Toxin
